# Development of Biodegradable Polymeric Stents for the Treatment of Cardiovascular Diseases

**DOI:** 10.3390/biom12091245

**Published:** 2022-09-06

**Authors:** Yihong Shen, Xiao Yu, Jie Cui, Fan Yu, Mingyue Liu, Yujie Chen, Jinglei Wu, Binbin Sun, Xiumei Mo

**Affiliations:** Shanghai Engineering Research Center of Nano-Biomaterials and Regenerative Medicine, College of Biological Science and Medical Engineering, Donghua University, Shanghai 201620, China

**Keywords:** cardiovascular disease, biodegradable polymeric stents, manufacturing method, functionalization

## Abstract

Cardiovascular disease has become the leading cause of death. A vascular stent is an effective means for the treatment of cardiovascular diseases. In recent years, biodegradable polymeric vascular stents have been widely investigated by researchers because of its degradability and clinical application potential for cardiovascular disease treatment. Compared to non-biodegradable stents, these stents are designed to degrade after vascular healing, leaving regenerated healthy arteries. This article reviews and summarizes the recent advanced methods for fabricating biodegradable polymeric stents, including injection molding, weaving, 3D printing, and laser cutting. Besides, the functional modification of biodegradable polymeric stents is also introduced, including visualization, anti-thrombus, endothelialization, and anti-inflammation. In the end, the challenges and future perspectives of biodegradable polymeric stents were discussed.

## 1. Introduction

Coronary artery disease (CAD), a disease that is characterized by the deposition of cholesterol, fats, calcium, and other substances in the arteries, has become the leading threat to public health [1,2]. As a characteristic of the CAD, a thrombus is formed in the flowing blood and blocks the artery. According to a WHO report, more than 60 million potential years of life are lost to vascular diseases in Europe annually [3]. In face of this high mortality disease, stent-based interventional surgery is one of the most popular therapeutic approaches for CAD. The application of cardiovascular stents can be deployed to the lesion site to “open” stenosis and restore patency of blood flow. 

Starting from the middle of the last century, interventional treatment has gradually developed. Charles Dotter performed the first percutaneous transluminal angioplasty using a catheter, making him the founder of interventional radiology [4]. Andreas Grüntzig performed the first successful percutaneous transluminal coronary angioplasty using an inflatable balloon dilatation catheter, ushering in a new era of coronary intervention [5]. After these pioneering works, percutaneous coronary intervention (PCI) has become one of the main treatments of vascular diseases. From the 1980s and 1990s, the original stents were mainly composed of bare metal stents which were made of stainless steel (SS) or cobalt-chromium (CoCr) alloy [6]. Bare metal stents can maintain strong a support force after implantation, thus preventing the restenosis of diseased vessels. However, it is easy to cause inflammation and intimal hyperplasia of diseased blood vessels [7], which leads to the high incidence of intravascular restenosis after implantation [8]. Therefore, in order to inhibit inflammation and intimal hyperplasia, an organic polymer layer is coated on the metal surface to prepare the drug-eluting stent, which contains drugs that can regulate inflammation and cell proliferation. Compared with bare metal stents, the drug-eluting stent can effectively inhibit intimal hyperplasia to prevent in-stent restenosis [9]. Nevertheless, drug-eluting stents did not reduce the actual mortality. The introduction of drugs will lead to adverse risks such as delayed arterial healing, late stent thrombosis, and delayed endothelialization [10]. In fact, although bare stents and drug-eluting stents can play a good role in vascular support in the early stage of implantation, their non degradability determines that long-term stents in the lumen are bound to bring inflammation and intimal hyperplasia, which will seriously reduce the clinical long-term patency rate. Moreover, it will accompany patients for their whole of life, which will affect the patient’s physical health and well-being.

Therefore, in order to avoid permanent retention in the body, it is meaningful to develop a biodegradable vascular stent that can support the blocked artery and restore normal blood flow after implantation [11]. After the degradation of biodegradable stents, the treated arteries can regain their normal elasticity to expand and contract [12,13]. Biodegradable stents can also reduce the risk of stent restenosis and stent thrombosis. Considering that it can be degraded into non-toxic small molecules, biodegradable stents can be considered as a promising approach in interventional cardiology [14]. In fact, biodegradable stents can be divided into two kinds: biodegradable metallic stents and biodegradable polymeric stents. The biodegradable metallic stents are mainly made of magnesium, iron, zinc, and their alloys. A wide range of synthesized polymers, such as poly-L-lactic acid (PLLA), poly (lactide-co-glycolic acid) (PLGA), poly glycolic acid (PGA), and poly-ε-caprolactone (PCL) have been extensively exploited for biodegradable polymeric stents. Compared to biodegradable metallic stents, synthetic polymers are easily tailored into various structures and shapes as needed for biodegradable stents. The selection of materials should be available for stent implantation and the specific requirements of various medical applications.

**Figure 1:**
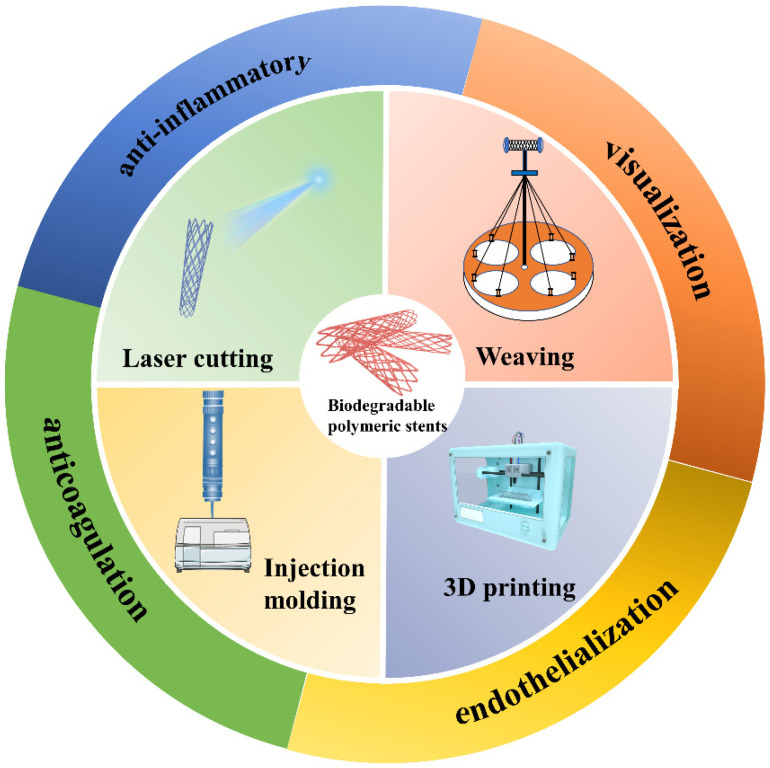
Current research progress in biodegradable polymeric stents.

In this review, we have briefly summarized the work of biodegradable polymeric stents that were prepared by various methods. Thereafter, we enumerate post-modification strategies to boost the performance of the stents (Figure 1). Furthermore, the future outlook in the development of biodegradable polymeric stents has also been discussed. 

## 2. Manufacturing Methods of Biodegradable Polymeric Stents

The biodegradable polymeric stent has been considered as a preferred choice for interventional therapy due to its degradability. Due to the small size of vascular stents, they are in strict demand for precision. Moreover, the properties of the stent material limit the use of different processing methods. The manufacturing method plays an important role in the stent properties and the success rate of implantation. Below, we evaluate the various methods for the preparation of biodegradable stents, such as laser cutting [15], injection molding [16], weaving [17], 3D printing [18], and so on (Table 1).

### 2.1. Injection Molding

The injection molding technique is a simple and versatile processing technique to prepare biodegradable polymeric stents. The mechanism of injection molding is to inject the thermoplastic material or thermosetting material into the desired vascular stent mold to prepare the vascular stent [19]. In this way, a large number of vascular stents with the same structure and specifications can be fabricated in a short time. This production method can also apply different materials, which shows a high degree of flexibility [20].

Liu et al. [21] used polycaprolactone (PCL) as a raw material and developed two types of biodegradable polymeric stents through a micro-injection molding machine. Different parts were assembled and welded into 3-5 mm vascular stents, which were balloon expandable and had a self-lock design. The stent could expand rapidly at five atmospheres, which is similar to the deployment pressure of metal stents. The special design of the stent element could effectively resist the external pressure of the blood vessels. During the degradation experiment, the compressive strength and collapse pressure of the PCL stents did not decrease significantly and remained stable. 

For the bifurcation vessel, Lee et al. [22] prepared PCL biodegradable bifurcated stents by injection molding and hot spot welding. The biodegradable bifurcated stent integrated the main vessel and two branches into one part, especially at the coronary artery anastomosis. The special design may prevent restenosis in the site of artery anastomosis. Moreover, the stent exhibited mechanical properties that were comparable to commercial metallic stents. The above design aimed to compositionally utilize injection molding to achieve a new idea for the treatment of bifurcated diseased blood vessels.

From the above cases, it is seen that the injection molding allows for the manufacture of biodegradable polymeric stents. However, the vascular stents are usually tiny in size and have many complicated structures, so that the injection molding stents are difficult to achieve high precision. At the same time, it is difficult to fill and demolding the stent in the process of preparation, which largely limits their application. Moreover, the complete consistency of structural size is also hard to meet the purpose of personalized medical treatment [23].

### 2.2. Laser Cutting

Laser cutting is another method for vascular stent preparation, which can be divided into two kinds. Amongst, the first one is to carve the desired plane shape on the polymer plate. The carved plate is crimped into a cylinder, and then both ends are melted and bonded to make vascular stents. The second approach is to use the polymer material to manufacture the tubular material. The required structure is carved on the polymeric tube by laser cutting. Finally, the vascular stent is cut off from the tube [24]. Different types of laser techniques can be used in the manufacture of stents, such as CO_2_ laser [25], Nd:YAG laser [26], fiber laser [27], excimer laser [28] and ultrashort pulse laser [29] and so on. The stent fabricated by laser technology has the advantages of high precision, which is widely concerned.

Stępak et al. [30] used a CO_2_ laser to cut 250 mm thick poly (L—lactide) (PLLA) and poly (lactide-co-glycolic acid) (PLGA) sheets to fabricate polymeric stents. After the optimization of the laser parameters, the thickness of the stent struts could be controlled within 300 μm. The mechanical property tests showed that the Young’s modulus and the ultimate tensile strength of the elements that were fabricated by CO_2_ laser were better than those that were fabricated by an excimer laser. The edge that is created by the CO_2_ laser may strengthen the mechanical property of the stent. Hence, the feasibility of fabricating stents by CO_2_ laser was confirmed.

In another method, Guerra et al. [31] explored the feasibility of manufacturing the vascular stents via a 1.08 μm wavelength fiber laser. The results showed the feasibility of cutting the PCL sheets, which could achieve the precision of more than 95.75% with a taper angle that was lower than 0.033°. As the energy density input that is produced by the laser was increased, the crystallinity percentage increased. Moreover, the fiber laser process had exhibited a minor effect on the mechanical properties of the polymer. This work showed the feasibility of a 1.08 μm wavelength fiber laser to make PCL stents.

While the laser cutting techniques have the above advantages, their applicability is still limited and restricted [32]. In the process of preparation, it is easy to cause thermal damage. Microcracks and stripes may occur on the surface of the struts, which affect the mechanical properties of the stent. Owing to the limitations of laser cutting, the surface of the stent strut may form sharp edges, which may damage blood vessels or lead to unstable blood flow after implantation. Besides, the disadvantages such as high cost, long time, and the lack of longitudinal flexibility should also be considered.

### 2.3. Weaving

As the traditional preparation technology for fabricating metallic stents, the weaving methods are also of great promise in the manufacture of biodegradable polymeric stents. According to the shape and size of the required stent, the designed structure of the stent can be directly woven by multiple strands of monofilament. On account of the flexibility and elasticity, braided stents can be easily delivered to the circuitous and narrowed blood vessels through the catheter during the operation [33]. At the same time, it also has good axial compliance, which makes it less likely to damage the blood vessel wall after implantation and is conducive to resume blood stream [34].

**Figure 2:**
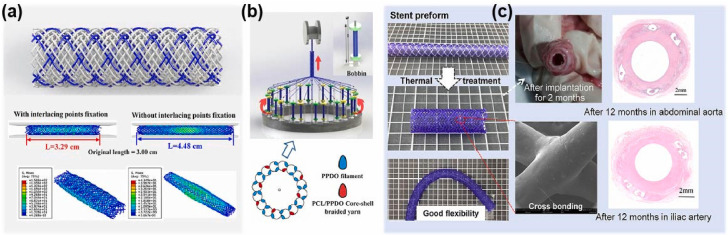
Design (**a**), processing (**b**), and implantation (**c**) of braided mechanical self-reinforcing composite stent.

The braided stents have been a hotspot of research in recent years. Zhao et al. [35] prepared bioresorbable poly (p-dioxanone) (PPDO) stents via the braiding technology. Then, the stents were thermally treated under different temperatures. It was found that thermal treatment improves the radial performance of PPDO braiding stents. However, the braided stents still exhibit unsatisfactory mechanical resistance, and tend to deform under pressure. Hence, Zhao et al. [36] further developed a mechanical self-reinforcing composite bioresorbable stent for congenital heart disease by combining braiding technology and an annealing process. The stent consisted of a PPDO and PCL/PPDO core-shell composite yarn (Figure 2). The intersecting points in the structure were partially bonded, so that the stent had higher compression force and good flexibility than the typical braided structure. In the implantation of the abdominal aorta and iliac artery of porcine, the stent was completely endothelialized within 1 month and showed excellent patency of target vessels during the 12-month follow-up.

**Figure 3:**
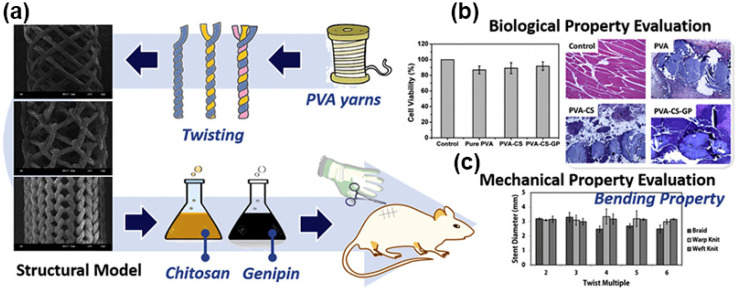
Schematic illustration for the formation and evaluation of a PVA stent. (**a**) The design and processing of the PVA stent. (**b**) The biological property evaluation of the PVA stent. (**c**) The mechanical property evaluation of the PVA stent.

In addition, the weaving method will affect the performance of the stents. Lin et al. [37] combined one, two, or three biodegradable polyvinyl alcohol (PVA) yarns with different twist factors and twists to form single, two, and three PVA twisted yarns. Braided, warp-knitted, and weft-knitted PVA vascular stents were prepared by PVA twisted yarns. Then, chitosan was coated to fix the intersecting point of the PVA vascular stent, and genipin was used for chemical cross-linking to enhance the mechanical properties of the vascular stent (Figure 3). The results showed that the stent had good bending resistance properties. Among them, the weft-knitted stent exhibited the best bending performance. The more twisting layers of the PVA stent, the better the stability in a wet environment. Moreover, the weft-knitted stent had a porous structure that allowed the exchange of nutrients and the discharge of metabolic wastes. It also provided sufficient space for cell migration and proliferation. It also remained in an intact formation after being embedded in vivo for 28 days. Consequently, the stent provided an ideal manufacturing method for the study of vascular stents.

Compared with the two methods that were introduced above, the weaving method is an easy-to-perform processing method which can achieve continuous production and processing [38,39]. Indeed, weaving is a promising approach for the industrial production of biodegradable polymeric stents. However, the radial force of weaving stents is relatively insufficient, which is difficult to meet the needs of long-term support in blood vessels. More research work is needed to elucidate its full potential and the limitations for biodegradable polymeric applications.

### 2.4. 3D Printing

Based on digital model files, 3D printing was proposed as a solution to achieve the personalized design for biodegradable polymeric stents. The desired structure can be prepared by adding layer upon layer 3D printing [40]. The digital model of diseased blood vessels is obtained through computed tomography (CT) and magnetic resonance imaging (MRI), which helps to achieve personalized implantation and other applications [41]. 3D printing stents can be designed according to the patient’s specific lesion environment and complex geometry to optimize the mechanical behavior, and then use it in the design of implants. The fabrication and development of tubular stents can be applied to 3D printing technologies such as fused deposition molding (FDM) [42], selective laser melting (SLM) [43], and stereo lithography appearance (SLA) [44]. A 3D printed vascular stent has its unique advantages. The cross-section of the printed-stent strut is circular, while the laser-cut stent is rectangular. Therefore, in the case of the same thickness of the stent strut, the circular stent strut has a smaller cross-sectional area, where the degradation rate in the body will be faster. At the same time, compared with laser cutting, the speed of using 3D printing technology to prepare vascular stents is faster, which takes little time to print a coronary stent [45].

**Figure 4:**
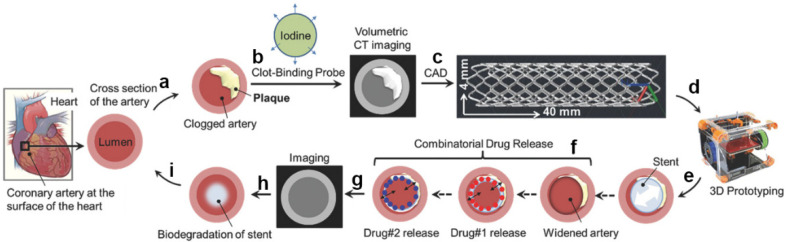
Stepwise process for developing a personalized coronary stent. (**a**) Cross-section of the artery shown at the surface of the heart, plaque buildup is shown inside the lumen due to various factors, e.g., aging, diet, genetics; (**b**) a clot-binding probe, i.e., a fibrin-targeted iodinated CT contrast probe is administered to locate the blood clot. At the same time, volumetric CT imaging helps to get an accurate measurement of the blockage; (**c**) the imaging information is transferred to a computer-aided design (CAD) software to design a personalized stent; (**d**) a PCL-GR polymer composite is used for fused deposition modeling additive manufacturing technique in a commercial 3D printing machine; (**e**) the prototyped stent is placed inside the artery; (**f**) the incorporation of two drugs for sequential release; (**g**) the healing process is further monitored by CT imaging; (**h,i**) the polymer gets biodegraded inside a widened artery.

Misra et al. [46] fabricated a flat stent by 3D printing, and then folded the flat stent to form a biodegradable PCL-graphene composite stent (PCL-GR stent) (Figure 4). Mechanical tests showed that the 3D printed stent exhibited excellent mechanical properties under deformation force. The introduction of graphene improved the resistance under pressure compared to the PCL stents. In vitro cell experiments showed that the stent had no obvious cytotoxicity. The ex vivo feasibility study in a porcine model also confirmed the possibility of implanting in patients after being improved in the future. However, the complicated post-treatment of a flat stent would reduce the fabrication efficiency. Hence, Zhao et al. [47] designed a self-made 3D printing system. The system consisted of a 3D printing system for fused deposition modeling and a rotating rod with controllable rotational speed. According to different models, the printing system could prepare different shapes of intravascular stents layer by layer under the requirements. The printed stent could be easily separated from the axis of rotation, which didn’t require complex subsequent processing. This preparation method could significantly improve the preparation efficiency and maintain the integrity of the stent.

**Figure 5:**
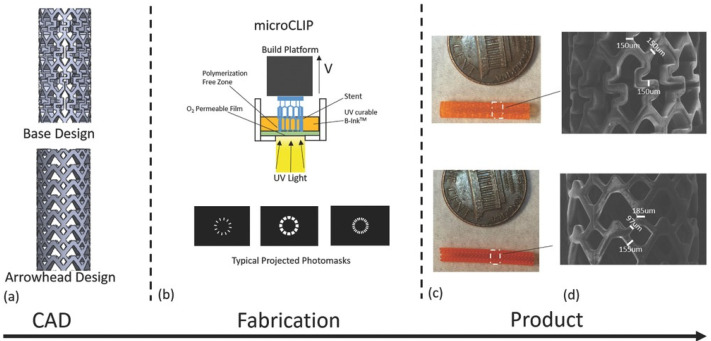
Stents can be printed with high design flexibility and resolution. (**a**) CAD images of the initial/primary design (base design) and a secondary design (arrowhead design) that were analyzed in this study. (**b**) Diagram of continuous liquid interface production microstereolithography (microCLIP) with typical projected photomasks of the stent. (**c**) 3D-printed base design (top) and arrowhead design (bottom) stents. (**d**) Scanning electron microscopy images of the base design (top) and arrowhead design (bottom).

For a self-expandable stent, Jia et al. [48] used a 3D printing technique to prepare biodegradable PLA vascular stents with self-expanding properties. The printed stent could be crimped into a temporary shape with a smaller diameter for implantation. The crimped stent had good shape solidity and could be stored in a temporary shape at room temperature. When implanted in the body, the shape memory PLA stent was thermally triggered to expand itself to its original shape under the influence of body temperature. The combination of the self-expanding properties of the biodegradable shape memory PLA and the personalized design of 3D printing provided a solution for the treatment of cardiovascular diseases in the future for vascular stents. In another study, Robert et al. [49] synthesized a multifunctional polydiolcitrate as the main component. The material was light-curable by incorporating methacrylate groups. The biodegradable stents were individually tailored through a micro-continuous liquid interface production system (microCLIP) and exposed to UV light for polymerization (Figure 5). At the same time, the stent had good mechanical properties. Deployment in vitro demonstrated that the stent could be compressed and self-expanded according to clinical requirements. The feasibility of implanting in the porcine artery was also confirmed.

As mentioned above, different preparation processes can be used to manufacture vascular stents with different characteristics. The comparison of biodegradable materials and preparation techniques for polymeric stents is shown in Table 2 below.

## 3. Functionalization of Biodegradable Polymeric Stents

**Figure 6:**
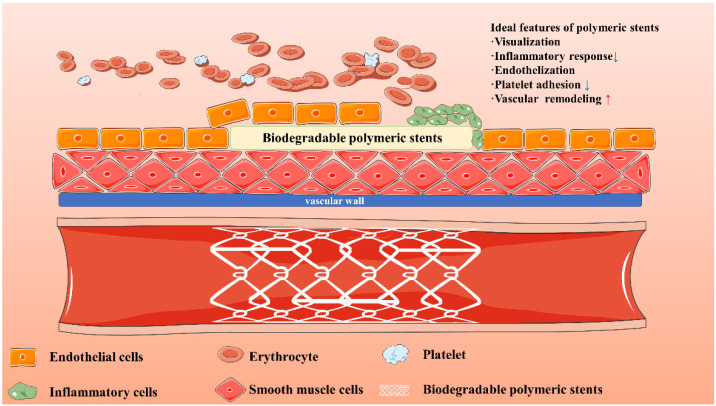
Ideal features of biodegradable polymeric stents. The features of stents including visualization, inflammatory response, endothelization, platelet adhesion, and vascular remodeling should be considered (red arrow↑ indicates the promotion effect, blue arrow↓ indicates the inhibition effect).

In order to adapt to clinical demand, the functionalization of biodegradable polymeric stents for cardiovascular diseases has also become the hot spot in the research and development field [50]. An ideal biodegradable polymeric stent should have the following characteristics (Figure 6): In the process of implantation, stents should be with the feature of visualization, which enable them to be accurately and safely deployed. After the implantation, the stent should prevent the adhesion of platelets. Moreover, the interaction between endothelial cells (ECs) and smooth muscle cells (SMCs) plays an important role in vascular repair after stent implantation. Ideal polymeric stents can promote the adhesion and proliferation of endothelial cells as well as suppress the excessive proliferation of smooth muscle cells [51]. The inflammatory responses around the stent site can also be inhibited. The functionalization of biodegradable polymeric stents is considered as the direction of the next generation of vascular stents.

### 3.1. Stent Visualization

The visualization of biodegradable stents is essential for positioning during the implantation and real-time monitoring [52]. This can effectively help clinicians understand the state of stents in deep tissue arteries. With the development of stent materials from metal to polymer, the visualization techniques of stents are also making continuous progress. Most polymer materials are composed of C, H, O, and N. They are elements with low electron density and specific gravity. Similarly, the human body is made up primarily of C, H, O, and N. In comparison to tissue, most polymers exhibit poor contrast and lack of radiopacity, so are unable to observe the real-time condition of the stent via X-ray imaging and MR imaging. It may have the clinical effects of failure that are associated with inability to access, deploy, or withdraw, which brings great difficulties for implantation. Moreover, it is difficult to assess the structural integrity, patency, and position of stents owing to the inability to monitor the stent during follow-up. Therefore, stents that are composed of polymers need to be visualized to avoid the above potential risks. Table 3 lists some of the commercial stent visualization methods.

As described in Table 3, the immobilization of radiopaque markers at the proximal and distal end of the stent is an important method for the visualization of commercial polymeric stents (except REVA Medical’s REVA and FANTOM stent) at present. However, these markers also have some disadvantages in clinical application [60]. The limitation of a radiopaque marker is that it cannot fully show the expansion of the stent because they are usually placed only at the distal and proximal ends. In addition, during the intraoperation and post-operation, clinicians will not be able to accurately evaluate the status of the stent, resulting in surgical difficulties [61]. Moreover, the degradation and morphological changes of stents should qualitatively or quantitatively be measured. The further treatment requirement can be determined by observing whether the cracks, defects, and morphological changes have occurred on the surface of stents. Therefore, the development of an entire visible stent has become a widespread concern of researchers.

In order to solve the problem of non-visualization of polymer materials, it may be effective to introduce some contrast agents with the polymer to increase the radiopacity of the stent. Common X-ray contrast agents, such as barium salt [62], iodine compound, and zirconia [63], can be introduced into the polymeric stents. To date, an array of techniques have been pursued for visualization: physical blend, surface coating, and chemical modification. These novel imaging techniques may bring new possibilities to show the expansion and position of the stent, which realize the comprehensive monitoring of the stent compared with the marker immobilization. 

**Figure 7:**
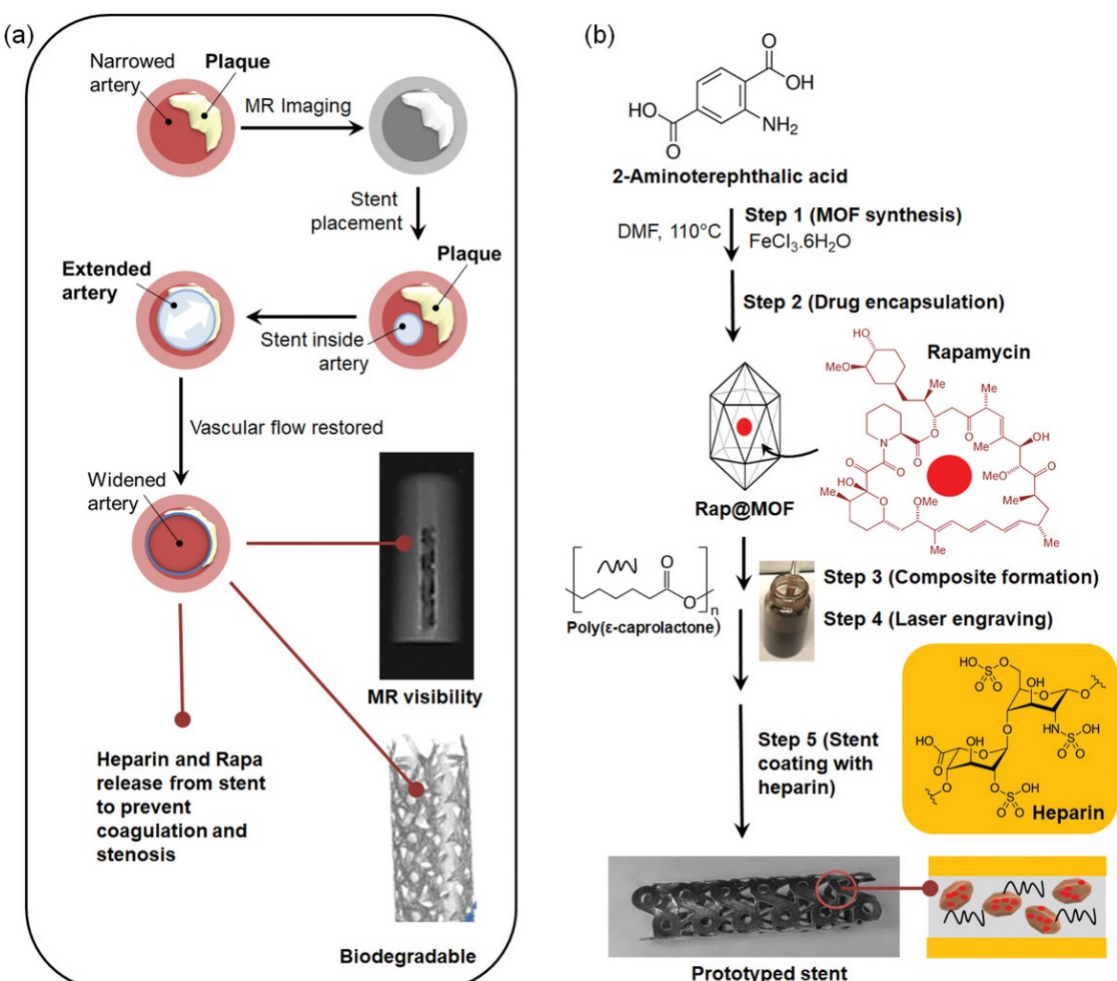
Schematics of MOF-composited polymeric stent: (**a**) Use of MOF-composited polymeric stent to prevent coagulation and stenosis while providing MRI visibility and biodegradability. (**b**) Stepwise process for the development of heparin-coated theranostic MOF-reinforced polymeric stents.

The combination of polymer matrix and contrast agent has been developed to achieve a robust method for the preparation of visible polymeric stents. Ang et al. [64] prepared a composite material that was based on inorganic BaSO_4_ nanofiller and PLLA. After being filled with BaSO_4_, the composite could be observed under X-ray. The larger the filling loading amount, the higher the radiopacity of the samples. Therefore, it could help to solve the limitations of visualization. In another study, Wang et al. [65] synthesized a new contrast agent: (S)-2-hydroxy-3-(4-iodobenzyloxy) propanoic acid. The structure of the compound was similar to L-lactic acid, which contained covalently-bound iodine. Blends of contrast agents and poly (D, L-lactic acid) were subsequently prepared by a micro-extrusion/blending method. The data showed that the intrinsic attenuation coefficient of (S)-2-hydroxy-3-(4-iodobenzyloxy) propanoic acid (the ability of the material to absorb X-rays) was stronger than that of commercial sodium diatrizoate. Therefore, it was possible to prepare completely bioabsorbable radiopaque coronary artery stents. X-ray visibility helped doctors ensure that the stent was fully expanded. These biodegradable blends had great potential applications in endovascular stents and other medical devices. For MRI visualization, Hamideh et al. [66] developed a metal organic framework (MOF)/PCL MRI-visible stent with a heparin coating. MOF was a crystalline porous material with network structure, which consisted of metal ions and organic ligands. The MOF material (NH_2_-MIL-101(Fe)) was encapsulated with rapamycin and mixed with PCL. Then, the composite was prepared into a thin sheet, and then was cut into a vascular stent by laser cutting and modified by heparinization (Figure 7). In vitro MRI imaging showed that the T2 contrast of these stents was significantly higher than that of invisible polymeric stents. This result was observed by T2* weighted gradient echo sequences, which made it possible to realize the visualization in vivo. The combination of MOF and polymer materials in this work may provide a potential possibility for the MRI visualization of polymeric stents. However, there are still some limitations of physical blending. The contrast agents are prone to aggregation, which largely limits the application of imaging.

As an attempt to prepare the visible coating, Lee et al. [67] applied the coating on the surface of the PLLA material. The coating was composed of 2, 3, 5-triiodobenzoic acid (TIBA), magnesium hydroxide (MH), and poly (d-L-lactic acid) (PDLLA). Among them, the introduction of TIBA increased the radiopacity. The radiopacity ability of the coating could be comparable to that of cobalt-chromium stents. Moreover, the in vitro release test showed that TIBA was released less than 10% for 28 days, which showed the stability of visible coating. Therefore, this coating may provide an idea for stent visualization. 

In addition to the method of physical blend, surface coating, the chemical modification of the polymer material is also an effective method to improve the visualization ability of the stent. Olsen et al. [68] developed a new biodegradable polyester, iodine poly(capro-lactone-co-1-4-oxepan-1,5-dione) (i-P(CLcoOPD)). O-(2-iodobenzyl) hydroxylamine was grafted onto P(CLcoOPD) to prepare i-P(CLcoOPD). The results showed that the functionalization with iodine was effective for X-ray contrast imaging. The imaging intensity could be quantified under different thicknesses of tissues (0.2–9 cm), so that the clinical tissue could be detected at different depths. At the same time, the degradation of polymer in vivo and in vitro could be quantified by radiography. Meanwhile, the changes of physical defects, such as cracks, could also be measured. In another work, Goodfriend et al. [69] synthesized radiopaque and MRI visible polymers- poly(gadodiamide fumaric acid) (PGFA). They used an MRI contrast agent, gadodiamide as a polymerization initiator for the synthesis of poly (propylene fumarate) (PPF). The application of this new contrast agent in medical devices was further evaluated. The addition of gadolinium could provide free radicals with four carboxylic acid groups. The production of free radicals initiated the transesterification reaction, which enabled poly-diamine to combine with the polymer chain to form a stable polymer. This unique polymer had good mixing uniformity with PLGA. The result showed that PLGA/PGFA nanoparticles have a high relaxation coefficient: r_1_ = 4.85, r_2_ = 1.33. This created a new paradigm for fully bioabsorbable medical devices with MRI visualization. Overall, with the continuous development of the stent, the chemical modification on the polymer may have great potential in the field of biodegradable stent visualization.

In clinical application, the realization of dual-mode visualization of X-ray and MRI has important clinical significance for polymeric stents. X-ray visualization can help clinicians locate stents during implantation, and MRI visualization can provide reference for follow-up work after stent implantation. Huang et al. [70] successfully synthesized uniform and monodisperse GdPO_4_·H_2_O nanowires by a solvothermal method. Then, the nano-bundles were compounded into HA/PLGA composites to obtain biodegradable and traceable polymer materials. GdPO_4_·H_2_O could be observed under both MRI and CT. When the concentration was greater than 0.66%, the MRI signal intensity increased with the increase of GdPO_4_·H_2_O concentration. Therefore, the combination of MRI and CT imaging could effectively monitor the condition of implants at different stages.

From the above cases, it is seen that the physical blend, surface coating, and chemical modification allow for the visualization of scaffolds biodegradable polymeric stents. By chemical or physical treatments, the biodegradable polymeric stents with visualization property can be observed in vitro, but the visualization of the polymeric stents in vivo remains a severe challenge, which requires more advanced design to solve.

### 3.2. Biological Functional Modification of Polymeric Stents

Stent thrombosis is one of the main reasons for the failure of stent implantation. The main causes of stent thrombosis are related to delayed endothelialization of the stent, excessive proliferation of vascular smooth muscle cells, and inflammatory responses. Therefore, it is beneficial to functionalize the polymeric stents by some bioactive cues. In this section, we focus on the modification of vascular stents and their biological significance (Table 4).

#### 3.2.1. The Anticoagulant and Endothelialization-Promoting Modification of Polymeric Stents

Generally, blood vessels are divided into three layers. The inner layer is tunica intima, which consists of the vascular endothelium and subendothelial layer. The middle layer is tunica media, which is mainly composed of SMCs and cytoplasmic matrix. The outer layer is tunica adventitia, which consists of fibroblasts and connective tissue. The endothelial cell layer of the tunica intima is easy to be damaged during surgery. After surgery, the subendothelial layer containing collagen and tissue factors is directly exposed to the blood, which is easy to cause the activation of platelets and coagulation system [78]. Therefore, the anticoagulation of the biodegradable stents is crucial for stent implantation.

**Figure 8:**
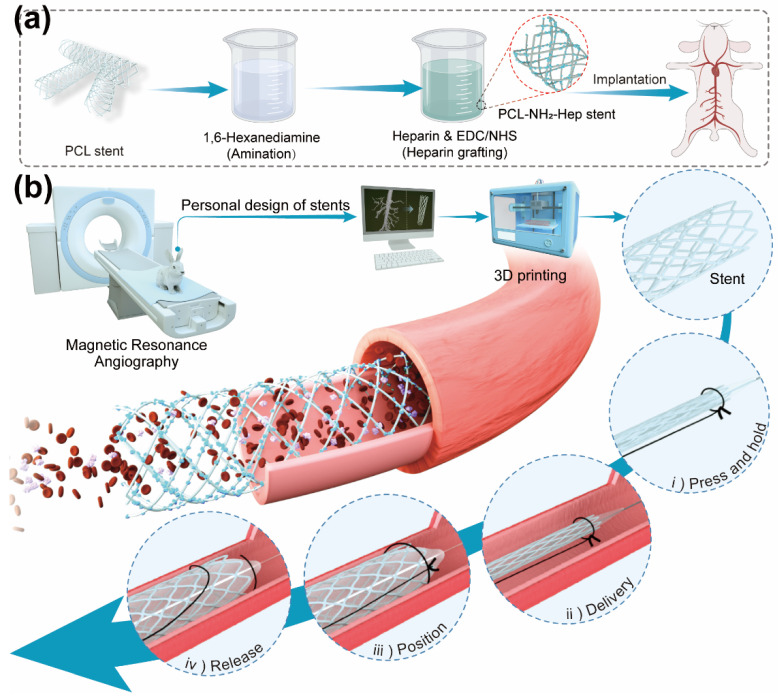
Schematic illustration for the preparation and implantation process of the heparinized PCL stent. (**a**) The process of heparinization modification of the PCL stent. (**b**) Schematic illustration of 3D printing personalized, anticoagulant, and biodegradable coronary artery stents guided by magnetic resonance angiography (MRA).

In order to improve the biological function of 3D printed stents, Shen et al. [71] used 3D printing technology to prepare PCL-based vascular stents, and then functionalized the stents with heparin by covalent grafting (Figure 8). The platelet adhesion test showed that the heparin covalently-modified stents had less platelet deposition compared to the PCL-based stents and would not cause platelet activation. The results of prothrombin time and activated partial thromboplastin time test were entirely coincided with the SEM figures of platelet adhesion. In the abdominal aorta of rabbits, the heparinized stent was completely endothelialized within 1 month and maintained good patency during the 3-month follow-up. Therefore, the stent with anticoagulant properties provides the possibility for the implantation in rabbit abdominal aorta.

Besides the basic requirements of anti-coagulation property, rapid endothelialization on the surface of vascular stents is considered as one of the core strategies to solve the formation of thrombus. The endothelial cells layers homogeneously cover on the surface of the stent, which indicates the completion of endothelialization process. Moreover, endothelial cells can adjust vascular repair and remodeling. Vascular smooth muscle cells (SMCs) are the main cellular components of the vascular wall. The disorder of SMCs proliferation and migration constitute the basic pathological characteristics of in-stent restenosis and intimal hyperplasia. ECs can secrete a variety of cytokines to act on SMCs, effectively inhibit the proliferation and migration of SMCs, and maintain the contractile state [78,79]. Lyu et al. [72] constructed a bifunctional biomimetic coating for the surface modification of cardiovascular stent materials by layer-by-layer grafting. Cu^2+^-chelated DOTA and hyaluronic acid (HA) were continuously immobilized on the plasma polyallylamine (PPAm) stent through water-phase amidation. Cu-DOTA could self-catalyze the production of nitric oxide (NO), and HA could mimic the endothelial glycocalyx functions. The antiplatelet test in vitro and the ex vivo antithrombogenic test showed that the dual-functional HA@DOTA-Cu coating in NO donor-supplemented medium exhibited the potent antiplatelet property. In addition, the dual-functional HA@DOTA-Cu supplied favorable microenvironment for ECs growth. The endothelial cell density average and migration distance also confirmed the promoted ECs migration. Moreover, HA@DOTA-Cu exhibited the inhibitory effect on SMCs. The results of long-term implantation showed that the coating could significantly inhibit intimal hyperplasia and stent restenosis. In another work, Yang et al. [73] explored a new recombinant human Type III collagen (hCOLIII), which didn’t have a platelet-binding site but retained its affinity for endothelial cells. hCOLIII and HA were deposited on the surface of PLA stent layer by layer to form a multi-layer coating imitating the extracellular matrix. The in vitro results showed that the multi-layer coating could promote the proliferation of ECs. The recombinant collagen and HA accelerated the endothelialization process. Moreover, the proliferation of SMCs was suppressed. The HA coating could let SMCs to differentiate into the contractile phenotype, while hCOLIII interacted with the cells via integrins. The stent was implanted into the abdominal aorta of rabbits and the regeneration was observed. The in vivo results showed that endothelialization was enhanced, the inflammatory response was inhibited, and intimal hyperplasia was also inhibited. 

Although some coatings containing bioactive agents have an effect on endothelial cell growth and intimal repair, the maintenance of bioactivity of biomolecules and the stability of the coating remain a great challenge. The harsh sterilizations or other factors would decrease the bioactivity of biomolecules. The special design for drug loading coating microstructure is an effective approach to save the bioactivity of bioactive agents. Wang et al. [74] developed a functional medical coating with heterogeneous structure by microphase separation and dissolution. Rapamycin was embedded in a dense drug coating at the bottom. The porous spongy top layer endowed the coating with controllable and rapid loading of vascular endothelial growth factor (VEGF), which preserved the bioactivity of VEGF. The biocompatibility in vitro test showed that the loaded VEGF could effectively improve the viability and proliferation of ECs. The VEGF regulated the proliferate behavior of ECs through ERK-TSC-mTOR signal pathway, which ensured that the loading of VEGF is an effective way to relieve the side effect of rapamycin to the endothelium. The introduction of VEGF was conducive to the recovery of the endothelium recovery after the impact of rapamycin. After implantation of coronary artery in porcine, VEGF/rapamycin-loaded stents showed the excellent positive effect of the recovery of the endothelium and the anti-hyperplasia of SMCs. The stent surface was covered with an intact intima, which proved the excellent regeneration of endothelium. Therefore, the hierarchical capillary coating design was a valid approach to promoting the regeneration of the endothelial layer while inhibiting in-stent restenosis.

Summarizing all the above research, it is indicated that most of the polymers are not conducive to ECs and SMCs adhesion and proliferation because of the lack of bioactive compounds. It limits the clinical application of biodegradable polymeric stents. The introduction of various drugs and bioactive molecules is also a promising approach for the improvement of the anticoagulant properties and endothelialization for biodegradable polymeric stents. However, sustained drug release and protection of biological activity of bioactive molecules cannot satisfy all the requirements of clinical applications. More research work is needed to work out.

#### 3.2.2. The Anti-Inflammatory Modification of Polymeric Stents

Stent implantation will cause mechanical damage to the intima and corresponding stimulation to the vessel wall as a foreign body. When endothelial cells are damaged, platelets are activated. Inflammatory cells begin to gather, infiltrate, and release inflammatory factors, such as Interleukin-1 (IL-1), Interleukin-6 (IL-6), and tumor necrosis factor-α (TNF-α), which play a pivotal role in the injury-induced inflammatory response and the atherosclerosis. Restenosis after stent implantation is closely related to the inflammatory reaction and platelet activation [80,81]. The functional modification of the stent would be favorable for the improvement of the anti-inflammatory and anti-thrombotic ability of the stent, so as to prevent the occurrence of restenosis.

**Figure 9:**
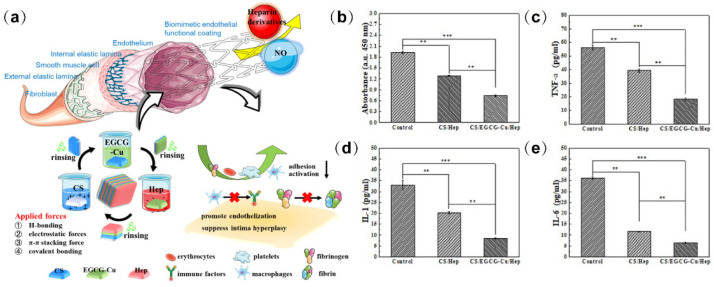
Illustration for the preparation and inflammation assessment of the sandwich-like LBL-coated stents (**a**) The coating that was fabricated with the sandwich-like LBL coating, in which chitosan, EGCG/Cu complex, and heparin was alternatingly assembled (**b**) Proliferation of macrophages that were incubated with samples for 24 h. The expression of TNF-α (**c**), IL-6 (**d**), and IL-1 (**e**). Statistical significance was evaluated using a one-way analysis of variance (ANOVA), ** *p* < 0.01, *** *p* < 0.001.

The polyphenols, as reactive substances with biological activity, are widely used. The anti-inflammatory characteristics of tannic acid, epigallocatechin gallate, and other polyphenols as natural antioxidants and their therapeutic potentials have been widely studied in the stent surface modifications. Qiu et al. [75] covalently immobilized the tannic acid (TA) and thrombin inhibitor bivalirudin (BVLD) through a phenolic-amine chemistry strategy. The behavior of macrophages on the TA/BVLD-modified stent surfaces showed that the expression of the inflammatory cytokine IL-6 was inhibited. The phenolic hydroxyl groups of TA suppressed the inflammatory response. The combined function of TA and BVLD could provide an ideal microenvironment for endothelialization and reduced intimal hyperplasia, which may be a promising strategy to solve the clinical complications that are related to restenosis and late stent thrombosis. In another study, Zhang et al. [76] developed a sandwich-like layer-by-layer coating using chitosan (CS) and heparin (Hep) as polyelectrolytes and embedding epigallocatechin gallate/copper (EGCG/Cu) complex (Figure 9). The incorporation of EGCG-Cu enhanced the stability of the coating. For the study of the inflammatory response in vitro, macrophages were cultured with samples. The results showed that less IL-1, IL-6, and TNF-α were released from the macrophages that were cultured with CS/EGCG-Cu/Hep group compared to the untreated surface. The subcutaneous implantation experiment in Sprague-Dawley rats also showed that no obvious invasive macrophages were observed at the CS/EGCG-Cu/Hep surfaces, indicating that the introduction of EGCG and heparin presented a synergistic effect on inflammatory responses. Therefore, the sandwich coating could be used as an effective surface modification strategy for cardiovascular implant devices to reduce the inflammatory responses of the stent.

Alternatively, zwitterionic polymer coatings with anti-inflammatory properties have been extensively studied. Yang et al. [77] synthesized zwitterionic poly (carboxybetaine acrylate–co–dopamine methacrylate) copolymer (PCBDA) that was inspired by mussel. Then, a zwitterionic polymer coating was formed on the surface of PLA stents by the co-deposition of poly (dopamine) (PDA) and polyethyleneimine (PEI). The assessment of the interaction of the samples with macrophages showed that that the PCBDA/PDA–PEI coating significantly inhibited the adhesion and the activation of macrophages because of its zwitterionic property. Moreover, the subcutaneous implantation experiment in Sprague-Dawley rats also showed that the PCBDA/PDA–PEI coating exhibited the mildest inflammatory response. The nonspecific protein adsorption was resisted by PCDBA, which mitigated the tissue response around the coating interface. In vivo experiments showed that the PCBDA/PDA-PEI coating could effectively inhibit intimal hyperplasia and tissue inflammation around vascular stents. Therefore, the coating provided an idea for polymeric stents to inhibit the tissue response and restenosis of stents. 

Conclusively, the utilization of the polyphenols and zwitterionic polymer is a valid method of for reducing the tissue response after biodegradable stent implantation.

## 4. Conclusions and Future Outlook

To sum up, this review summarizes the current application and development of polymeric stents in preclinical research. At present, some research results have shown gratifying results. However, this is only the beginning of the era of biodegradable polymeric stents for cardiovascular diseases. An ideal stent should have good mechanical properties and keep the stent stable before the formation of intima in neovascularization wall. Different techniques such as weaving, 3D printing, laser cutting, and injection molding can be selected according to different needs. During and after surgery, the stent should be visible and convenient for clinicians to observe and follow up. The visualization of the entire stent is one of the development trends of stent polymeric stents in the future. At the same time, polymeric stents should have good biocompatibility and have the promotion effect on endothelial cell viability, adhesion, proliferation, and migration. Different drug coatings can locally deliver anti-coagulant or anti-inflammatory drugs, which can adjust the state of diseased blood vessels and restore them to the normal state. Although the biodegradable polymeric stent has various advantages, it still faces the following challenges.

First, compared to metallic stents, the biodegradable polymeric stents exhibit insufficient mechanical strength. To feed the demand of radical strength that is similar to metallic stents, the thickness of the polymeric stent strut is higher, which may hinder the progress of tissue regeneration and vascular healing. Moreover, the increased stent strut thickness will decrease the axial flexibility, which limits the delivery ability of the biodegradable stents.

Secondly, the degradation period of the stent should be in accord with vascular remodeling. Vascular remodeling can be divided into three stages. In the early stages (0-3 months) and middle stages (3–6 months), the implanted biodegradable stents should exhibit excellent mechanical properties, which could withstand the pressure that is exerted by the vessel wall and prevent acute elastic recoil of the artery. In the late restoration stage (6–12 months), the mechanical properties should gradually decrease and restore the function of the blood vessels.

Finally, the implantation method requires specific design for the stent, which enables the uniform deformation at the diseased site.

The selection of the stent material and stent structural design may have the potential to solve the problem. Ultimately, we believe that the further development of polymeric stents offers great hope for interventional therapy and improve the quality of patient’s lives with cardiovascular disease.

## Figures and Tables

**Table 1:** Comparison among manufacturing methods of biodegradable polymeric stents.

Method	Mechanism	Advantages	Disadvantages
Injection molding	Injecting thermoplastic materials or thermosetting materials into the molds	- Convenient in preparation techniques - Great flexibility	- Poor accuracy - Long cycle of fabrication - Difficult to demold
Weaving	Weaving the stent directly according to the shape and size via knitting, warp knitting, weft knitting, or other weaving technology.	- High flexibility and elasticity - Great longitudinal compliance - Easy to process	- Low radial support force
Laser cutting	Carving the required structure on the polymer material via laser and cutting the stent from the tube.	- High precision	- Thermal damage - Sharp edge of struts - High cost - Long fabrication time
3D printing	Layer-by-layer printing based on digital model	- Personal design - High efficiency	- Low precision

**Table 2:** Summary of injection molding, weaving, laser cutting, and 3D printing.

Polymer	Fabrication Method	Brief Description	Advantages	Ref
PCL	Injection molding	Diameter 3–5 mm PLGA/paclitaxel coating Self-lock design	Ideal mechanical strength Sustainable release of paclitaxel	Liu et al. [21]
PCL	Injection molding	Bifurcation design PLGA/sirolimus coating	Design for bifurcation lesions Comparable mechanical properties with those of commercial stents SMCs proliferation↓	Lee et al. [22]
PVA	Braiding warp knitting weft knitting	CS coating /Genipin chemical cross-linking	Good bending resistance property Antibacterial Satisfactory biological properties	Lin et al. [37]
PPDO/PCL	Braiding	Diameter 8 mm Consisted of PPDO monofilaments and PCL/PPDO core-shell composite yarns	Two-stage degradation Good structure stability Implantation in vivo in 12 months	Zhao et al. [36]
PLLA/PLGA	CO_2_ laser cutting	/	Applied for fabrication of some types of polymers Obtained a smooth and narrow cut in a 250 mm-thick polymer sheet	Stępak et al. [30]
PCL/PLA	Fiber laser cutting	/	High accuracy Less effect over the material properties	Guerra et al. [31]
PCL-graphene	3D printing	Patient-specific stenting process Dual drug incorporation	Good mechanical properties Low toxicity Personal design	Misra et al. [46]
multifunctional polydiolcitrate (mPDC)	microCLIP	Polydiolcitrates can be engineered to be light-curable by incorporating methacrylate groups	Comparable mechanical strength of nitinol stents Customizable, compressed, and self-expanded within a clinically relevant time frame upon deployment in vitro	Robert et al. [49]

**Table 3:** The introduction of some commercial stent visualization methods.

Stent	Company	Polymer	Marker Material Used	Visualization Method	Ref
REVA	Reva Medical	desaminotyrosine polycarbonate polymer (PTD-PC)	Iodized polymer	Iodine impregnation	[53]
Absorb 1.0	Abbott	PLLA	Platinum	Marker fixation	[54]
Fantom	Reva Medical	PTD-PC	Iodized polymer	Iodine impregnation	[55]
XINSORB	Shandong Hua’an Biotechnology	PLLA	Platinum	Marker fixation	[56]
Igaki-Tamai	Kyoto Medical	PLLA	Gold	Marker fixation	[57]
DESolve	Elixir Medical	PLLA	Platinum	Marker fixation	[58]
NeoVas	Lepu Medical	PLLA	Platinum	Marker fixation	[59]

**Table 4:** Summary of biological modification of biodegradable stents.

Biomaterial Category	Bioactive Cue	Biological Evaluation	Key Findings	Ref.
PCL	Heparin covalent grafting	In vitro and implantation	Platelet adhesion↓ SMCs proliferation↓ ECs proliferation↑	Shen et al. [71]
Plasma polymeric allylamine (PPAm)	Cu-DOTA and HA coating	In vitro and implantation	Thrombus formation↓ SMCs migration↓ ECs adhesion↑	Lyu et al. [72]
PLLA	hCOLIII and HA coating	In vitro and implantation	SMCs proliferation↓ ECs proliferation↑ Inflammation↓	Yang et al. [73]
PLLA	Rapamycin-VEGF Hierarchical coating	In vitro and implantation	ECs proliferation↑ SMCs proliferation↑	Wang et al. [74]
PPAm	TA and BVLD coating	In vitro and implantation	Inflammation↓ Thrombus formation↓	Qiu et al. [75]
PLLA	CS/EGCG-Cu/Hep coating	In vitro and implantation	Thrombus formation↓ IL-1, IL-6, and TNF-α expression↓	Zhang et al. [76]
PLLA	PCBDA coating	In vitro and implantation	Platelet adhesion↓ Fibrinogen adhesion↓ Inflammation↓	Yang et al. [77]

## Data Availability

No applicable.

## References

[1] Libby P., Theroux P. (2005). Pathophysiology of coronary artery disease. Circulation.

[2] Okrainec K., Banerjee D.K., Eisenberg M.J. (2004). Coronary artery disease in the developing world. Am. Heart J..

[3] Townsend N., Kazakiewicz D., Lucy Wright F., Timmis A., Huculeci R., Torbica A., Gale C.P., Achenbach S., Weidinger F., Vardas P. (2022). Epidemiology of cardiovascular disease in Europe. Nat. Rev. Cardiol..

[4] Barton M., Grüntzig J., Husmann M., Rösch J. (2014). Balloon angioplasty—The legacy of Andreas Grüntzig, MD (1939–1985). Front. Cardiovasc. Med..

[5] Grüntzig A.R., Senning Å., Siegenthaler W.E. (1979). Nonoperative dilatation of coronary-artery stenosis: Percutaneous transluminal coronary angioplasty. N. Engl. J. Med..

[6] Hanawa T. (2009). Materials for metallic stents. J. Artif. Organs.

[7] Weintraub W.S. (2007). The pathophysiology and burden of restenosis. Am. J. Cardiol..

[8] Toutouzas K., Colombo A., Stefanadis C. (2004). Inflammation and restenosis after percutaneous coronary interventions. Eur. Heart J..

[9] Inoue T., Croce K., Morooka T., Sakuma M., Node K., Simon D.I. (2011). Vascular inflammation and repair: Implications for re-endothelialization, restenosis, and stent thrombosis. JACC Cardiovasc. Interv..

[10] Joner M., Finn A.V., Farb A., Mont E.K., Kolodgie F.D., Ladich E., Kutys R., Skorija K., Gold H.K., Virmani R. (2006). Pathology of drug-eluting stents in humans: Delayed healing and late thrombotic risk. J. Am. Coll. Cardiol..

[11] Zhu Y., Yang K., Cheng R., Xiang Y., Yuan T., Cheng Y., Sarmento B., Cui W. (2017). The current status of biodegradable stent to treat benign luminal disease. Mater. Today.

[12] Commandeur S., van Beusekom H.M., van der Giessen W.J. (2006). Polymers, drug release, and drug-eluting stents. J. Interv. Cardiol..

[13] Pauck R., Reddy B. (2015). Computational analysis of the radial mechanical performance of PLLA coronary artery stents. Med. Eng. Phys..

[14] Hou L.-D., Li Z., Pan Y., Sabir M., Zheng Y.-F., Li L. (2016). A review on biodegradable materials for cardiovascular stent application. Front. Mater. Sci..

[15] Momma C., Knop U., Nolte S. (1999). Laser cutting of slotted tube coronary stents—State-of-the-art and future developments. Prog. Biomed. Res..

[16] Ang H.Y., Bulluck H., Wong P., Venkatraman S.S., Huang Y., Foin N. (2017). Bioresorbable stents: Current and upcoming bioresorbable technologies. Int. J. Cardiol..

[17] Laasch H.-U. (2013). Current designs of self-expanding stents. Self-Expandable Stents in the Gastrointestinal Tract.

[18] Domínguez-Robles J., Diaz-Gomez L., Utomo E., Shen T., Picco C.J., Alvarez-Lorenzo C., Concheiro A., Donnelly R.F., Larrañeta E. (2021). Use of 3D Printing for the Development of Biodegradable Antiplatelet Materials for Cardiovascular Applications. Pharmaceuticals.

[19] Polanec B., Kramberger J., Glodež S. (2020). A review of production technologies and materials for manufacturing of cardiovascular stents. Adv. Prod. Eng. Manag..

[20] Li H., Liu K., Zhao D., Wang M., Li Q., Hou J. (2018). Multi-objective optimizations for microinjection molding process parameters of biodegradable polymer stent. Materials.

[21] Liu S.J., Chiang F.J., Hsiao C.Y., Kau Y.C., Liu K.S. (2010). Fabrication of balloon-expandable self-lock drug-eluting polycaprolactone stents using micro-injection molding and spray coating techniques. Ann. Biomed. Eng..

[22] Lee C.H., Chen C.J., Liu S.J., Hsiao C.Y., Chen J.K. (2012). The development of novel biodegradable bifurcation stents for the sustainable release of anti-proliferative sirolimus. Ann. Biomed. Eng..

[23] Jiang W., Zhao W., Zhou T., Wang L., Qiu T. (2022). A Review on Manufacturing and Post-Processing Technology of Vascular Stents. Micromachines.

[24] Lin S.Y., Chen C.S., Chen Y.S., Ma S.F. (2014). Developing in Biodegradable Stents Technology. Key Eng. Mater..

[25] Tseng S.-F., Hung T.-Y., Chang C.-M. (2022). Mechanical and microstructural properties of additively manufactured Ti–6Al–4 V stents with CO_2_ laser postannealing treatment. Int. J. Adv. Manuf. Technol..

[26] Alefelder J., Philipp J., Engelmann U., Senge T. (1991). Stented laser-welded vasovasostomy in the rat: Comparison of Nd: YAG and CO_2_ lasers. J. Reconstr. Microsurg..

[27] Meng H., Liao J., Zhou Y., Zhang Q. (2009). Laser micro-processing of cardiovascular stent with fiber laser cutting system. Opt. Laser Technol..

[28] Badr S., Ben-Dor I., Dvir D., Barbash I.M., Kitabata H., Pendyala L.K., Loh J.P., Torguson R., Pichard A.D., Waksman R. (2013). The state of the excimer laser for coronary intervention in the drug-eluting stent era. Cardiovasc. Revasc. Med..

[29] Hendricks F., Patel R., Matylitsky V. Micromachining of bio-absorbable stents with ultra-short pulse lasers. Proceedings of the Frontiers in Ultrafast Optics: Biomedical.

[30] Stępak B., Antończak A.J., Bartkowiak-Jowsa M., Filipiak J., Pezowicz C., Abramski K.M. (2014). Fabrication of a polymer-based biodegradable stent using a CO_2_ laser. Arch. Civ. Mech. Eng..

[31] Guerra A.J., Farjas J., Ciurana J. (2017). Fibre laser cutting of polycaprolactone sheet for stents manufacturing: A feasibility study. Opt. Laser Technol..

[32] McClean D.R., Eigler N.L. (2003). Stent design: Implications for restenosis. Rev. Cardiovasc. Med..

[33] Kelly N., McGrath D.J., Sweeney C.A., Kurtenbach K., Grogan J.A., Jockenhoevel S., O’Brien B.J., Bruzzi M., McHugh P.E. (2019). Comparison of computational modelling techniques for braided stent analysis. Comput. Methods Biomech. Biomed. Eng..

[34] Venkatraman S., Boey F., Lao L.L. (2008). Implanted cardiovascular polymers: Natural, synthetic and bio-inspired. Prog. Polym. Sci..

[35] Zhao F., Xue W., Wang F., Yu C., Xu H., Hao Y., Wang L. (2017). A new approach to improve the local compressive properties of PPDO self-expandable stent. J. Mech. Behav. Biomed. Mater..

[36] Zhao F., Sun J., Xue W., Wang F., King M.W., Yu C., Jiao Y., Sun K., Wang L. (2021). Development of a polycaprolactone/poly(p-dioxanone) bioresorbable stent with mechanically self-reinforced structure for congenital heart disease treatment. Bioact. Mater..

[37] Lin M.C., Lou C.W., Lin J.Y., Lin T.A., Chen Y.S., Lin J.H. (2018). Biodegradable Polyvinyl Alcohol Vascular Stents: Structural Model and Mechanical and Biological Property Evaluation. Mater. Sci. Eng. C Mater. Biol. Appl..

[38] King M.W., Gupta B.S., Guidoin R. (2013). Biotextiles as Medical Implants.

[39] Jiang C., Wang K., Liu Y., Zhang C., Wang B. (2021). Application of textile technology in tissue engineering: A review. Acta Biomater..

[40] Shahrubudin N., Lee T.C., Ramlan R. (2019). An overview on 3D printing technology: Technological, materials, and applications. Procedia Manuf..

[41] Khoo Z.X., Teoh J.E.M., Liu Y., Chua C.K., Yang S., An J., Leong K.F., Yeong W.Y. (2015). 3D printing of smart materials: A review on recent progresses in 4D printing. Virtual Phys. Prototyp..

[42] Dudek P. (2013). FDM 3D printing technology in manufacturing composite elements. Arch. Metall. Mater..

[43] Yang Z., Yu Y., Wei Y., Huang C. (2017). Crushing behavior of a thin-walled circular tube with internal gradient grooves fabricated by SLM 3D printing. Thin-Walled Struct..

[44] Ma X.L. (2013). Research on application of SLA technology in the 3D printing technology. Appl. Mech. Mater..

[45] Giannopoulos A.A., Mitsouras D., Yoo S.-J., Liu P.P., Chatzizisis Y.S., Rybicki F.J. (2016). Applications of 3D printing in cardiovascular diseases. Nat. Rev. Cardiol..

[46] Misra S.K., Ostadhossein F., Babu R., Kus J., Tankasala D., Sutrisno A., Walsh K.A., Bromfield C.R., Pan D. (2017). 3D-Printed Multidrug-Eluting Stent from Graphene-Nanoplatelet-Doped Biodegradable Polymer Composite. Adv. Healthc. Mater..

[47] Zhao D., Zhou R., Sun J., Li H., Jin Y. (2019). Experimental study of polymeric stent fabrication using homemade 3D printing system. Polym. Eng. Sci..

[48] Jia H., Gu S.-Y., Chang K. (2018). 3D printed self-expandable vascular stents from biodegradable shape memory polymer. Adv. Polym. Technol..

[49] van Lith R., Baker E., Ware H., Yang J., Farsheed A.C., Sun C., Ameer G. (2016). 3D-Printing Strong High-Resolution Antioxidant Bioresorbable Vascular Stents. Adv. Mater. Technol..

[50] Tian H., Tang Z., Zhuang X., Chen X., Jing X. (2012). Biodegradable synthetic polymers: Preparation, functionalization and biomedical application. Prog. Polym. Sci..

[51] Su S., Kang P.M. (2020). Systemic review of biodegradable nanomaterials in nanomedicine. Nanomaterials.

[52] Attia M.F., Brummel B.R., Lex T.R., Van Horn B.A., Whitehead D.C., Alexis F. (2018). Recent Advances in Polyesters for Biomedical Imaging. Adv. Healthc. Mater..

[53] Waksman R. (2006). Update on bioabsorbable stents: From bench to clinical. J. Interv. Cardiol..

[54] Ormiston J.A., Serruys P.W., Regar E., Dudek D., Thuesen L., Webster M.W., Onuma Y., Garcia-Garcia H.M., McGreevy R., Veldhof S. (2008). A bioabsorbable everolimus-eluting coronary stent system for patients with single de-novo coronary artery lesions (ABSORB): A prospective open-label trial. Lancet.

[55] Chevalier B., Abizaid A., Carrié D., Frey N., Lutz M., Weber-Albers J., Dudek D., Weng S.-C., Akodad M., Anderson J. (2019). Clinical and angiographic outcomes with a novel radiopaque sirolimus-eluting bioresorbable vascular scaffold: The FANTOM II study. Circ. Cardiovasc. Interv..

[56] Shen L., Wu Y., Ge L., Zhang Y., Wang Q., Qian J., Qiu Z., Ge J. (2017). A head to head comparison of XINSORB bioresorbable sirolimus-eluting scaffold versus metallic sirolimus-eluting stent: 180 days follow-up in a porcine model. Int. J. Cardiovasc. Imaging.

[57] Nishio S., Kosuga K., Igaki K., Okada M., Kyo E., Tsuji T., Takeuchi E., Inuzuka Y., Takeda S., Hata T. (2012). Long-term (>10 years) clinical outcomes of first-in-human biodegradable poly-l-lactic acid coronary stents: Igaki-Tamai stents. Circulation.

[58] Abizaid A., Costa R.A., Schofer J., Ormiston J., Maeng M., Witzenbichler B., Botelho R.V., Costa J.R., Chamié D., Abizaid A.S. (2016). Serial multimodality imaging and 2-year clinical outcomes of the novel DESolve novolimus-eluting bioresorbable coronary scaffold system for the treatment of single de novo coronary lesions. JACC Cardiovasc. Interv..

[59] Han Y., Xu B., Fu G., Wang X., Xu K., Jin C., Tao L., Li L., Hou Y., Su X. (2018). A randomized trial comparing the NeoVas sirolimus-eluting bioresorbable scaffold and metallic everolimus-eluting stents. JACC Cardiovasc. Interv..

[60] Nagy P. (2015). X-ray examination of integrated stent markers. IRBM.

[61] Nagy P. (2015). X-ray Analysis of Stents and their Markers. Period. Polytech. Mech. Eng..

[62] Hong S.H., Herman A.M., Stephenson J.M., Wu T., Bahadur A.N., Burns A.R., Marrelli S.P., Wythe J.D. (2020). Development of barium-based low viscosity contrast agents for micro CT vascular casting: Application to 3D visualization of the adult mouse cerebrovasculature. J. Neurosci. Res..

[63] Kashyap D., Kumar P.K., Kanagaraj S. (2018). 4D printed porous radiopaque shape memory polyurethane for endovascular embolization. Addit. Manuf..

[64] Ang H.Y., Toong D., Chow W.S., Seisilya W., Wu W., Wong P., Venkatraman S.S., Foin N., Huang Y. (2018). Radiopaque Fully Degradable Nanocomposites for Coronary Stents. Sci. Rep..

[65] Wang Y., van den Akker N.M., Molin D.G., Gagliardi M., van der Marel C., Lutz M., Knetsch M.L., Koole L.H. (2014). A nontoxic additive to introduce x-ray contrast into poly(lactic acid). Implications for transient medical implants such as bioresorbable coronary vascular scaffolds. Adv. Healthc. Mater..

[66] Hamideh R.A., Akbari B., Fathi P., Misra S.K., Sutrisno A., Lam F., Pan D. (2020). Biodegradable MRI Visible Drug Eluting Stent Reinforced by Metal Organic Frameworks. Adv. Healthc. Mater..

[67] Lee H.I., Heo Y., Baek S.W., Kim D.S., Song D.H., Han D.K. (2021). Multifunctional Biodegradable Vascular PLLA Scaffold with Improved X-ray Opacity, Anti-Inflammation, and Re-Endothelization. Polymer.

[68] Olsen T.R., Davis L.L., Nicolau S.E., Duncan C.C., Whitehead D.C., Van Horn B.A., Alexis F. (2015). Non-invasive deep tissue imaging of iodine modified poly (caprolactone-co-1-4-oxepan-1, 5-dione) using X-ray. Acta Biomater..

[69] Goodfriend A.C., Welch T.R., Nguyen K.T., Wang J., Johnson R.F., Nugent A., Forbess J.M. (2015). Poly (gadodiamide fumaric acid): A bioresorbable, radiopaque, and MRI-visible polymer for biomedical applications. ACS Biomater. Sci. Eng..

[70] Huang J., Lv Z., Wang Y., Wang Z., Gao T., Zhang N., Guo M., Zou H., Zhang P. (2016). In Vivo MRI and X-Ray Bifunctional Imaging of Polymeric Composite Supplemented with GdPO_4_·H_2_O Nanobundles for Tracing Bone Implant and Bone Regeneration. Adv. Healthc. Mater..

[71] Shen Y., Tang C., Sun B., Zhang Y., Sun X., El-Newehy M., El-Hamshary H., Morsi Y., Gu H., Wang W. (2022). 3D printed personalized, heparinized and biodegradable coronary artery stents for rabbit abdominal aorta implantation. Chem. Eng. J..

[72] Lyu N., Du Z., Qiu H., Gao P., Yao Q., Xiong K., Tu Q., Li X., Chen B., Wang M. (2020). Mimicking the Nitric Oxide-Releasing and Glycocalyx Functions of Endothelium on Vascular Stent Surfaces. Adv. Sci..

[73] Yang L., Wu H., Lu L., He Q., Xi B., Yu H., Luo R., Wang Y., Zhang X. (2021). A tailored extracellular matrix (ECM)—Mimetic coating for cardiovascular stents by stepwise assembly of hyaluronic acid and recombinant human type III collagen. Biomaterials.

[74] Wang J., Xue Y., Liu J., Hu M., Zhang H., Ren K., Wang Y., Ji J. (2020). Hierarchical Capillary Coating to Biofunctionlize Drug-Eluting Stent for Improving Endothelium Regeneration. Research.

[75] Qiu H., Tu Q., Gao P., Li X., Maitz M.F., Xiong K., Huang N., Yang Z. (2021). Phenolic-amine chemistry mediated synergistic modification with polyphenols and thrombin inhibitor for combating the thrombosis and inflammation of cardiovascular stents. Biomaterials.

[76] Zhang B., Yao R., Hu C., Maitz M.F., Wu H., Liu K., Yang L., Luo R., Wang Y. (2020). Epigallocatechin gallate mediated sandwich-like coating for mimicking endothelium with sustained therapeutic nitric oxide generation and heparin release. Biomaterials.

[77] Yang L., Wu H., Liu Y., Xia Q., Yang Y., Chen N., Yang M., Luo R., Liu G., Wang Y. (2022). A robust mussel-inspired zwitterionic coating on biodegradable poly(L-lactide) stent with enhanced anticoagulant, anti-inflammatory, and anti-hyperplasia properties. Chem. Eng. J..

[78] Jeong Y., Yao Y., Yim E.K.F. (2020). Current understanding of intimal hyperplasia and effect of compliance in synthetic small diameter vascular grafts. Biomater. Sci..

[79] Jaminon A., Reesink K., Kroon A., Schurgers L. (2019). The Role of Vascular Smooth Muscle Cells in Arterial Remodeling: Focus on Calcification-Related Processes. Int. J. Mol. Sci..

[80] Welt F.G., Rogers C. (2002). Inflammation and restenosis in the stent era. Arterioscler. Thromb. Vasc. Biol..

[81] Drachman D.E., Simon D.I. (2005). Inflammation as a mechanism and therapeutic target for in-stent restenosis. Curr. Atheroscler. Rep..

